# Case report: Fluctuating tumor markers in a boy with gonadotropin-releasing hormone-independent precocious puberty induced by a pineal germ cell tumor

**DOI:** 10.3389/fped.2022.940656

**Published:** 2022-08-23

**Authors:** Alessandro Cattoni, Assunta Albanese

**Affiliations:** ^1^Department of Pediatrics, The Royal Marsden NHS Foundation Trust, London, United Kingdom; ^2^Department of Pediatrics, Fondazione MBBM, San Gerardo Hospital, University of Milano-Bicocca, Monza, Italy

**Keywords:** germ cell and embryonal neoplasms, precocious puberty, chorionic gonadotropin, pineal cyst, anastrozole, aromatase inhibitors, bicalutamide

## Abstract

GnRH-independent precocious puberty (GIPP) can be the presenting clinical picture experienced by patients with secreting germ cell tumor (GCT). Indeed, as luteinizing hormone (LH) and human chorionic gonadotropin (hCG) share identical α-subunits and similar β-subunits, an increased secretion of β-hCG may result in a precocious activation of Leydig cells. Though the co-occurrence of raised β-hCG levels and signs of precocious virilization usually prompts a complete oncological work-up, the diagnostic and therapeutic management of GCT-induced GIPP may be challenging. We report the case of a 6.2 year-old boy presenting with clinical and biochemical findings consistent with GIPP (discrepancy between overt virilization and pre-pubertal testicular volume, suppressed gonadotropins and remarkably raised testosterone). Brain imaging detected a bilobed cyst of the pineal gland, while serum and cerebrospinal baseline assessment initially ruled out raised alpha-fetoprotein or β-hCG levels. Nevertheless, a strict biochemical follow-up highlighted a fluctuant trend of tumor markers, with a more aggressive behavior and recurrent erections occurring as a result of unpredictable phases of raised testosterone and serum/cerebrospinal β-hCG, followed by sudden spontaneous decrease. Accordingly, a secreting pineal GCT was suspected. Given the fluctuating trend of tumor markers, surgery was initially kept on hold and a combined treatment with bicalutamide (androgen receptor blocker) and anastrozole (aromatase inhibitor) was undertaken in order to prevent the patient from experiencing further virilization and excessive bone age maturation. Subsequently, a progression in the size of the pineal tumor prompted surgical resection and a diagnosis of secreting GCT was histologically confirmed. Accordingly, the patient was started on adjuvant chemo- and radiotherapy. Antineoplastic treatment was followed by persistent and remarkable decrease of tumor markers and by a complete pubertal arrest. We reported the challenging diagnosis of a secreting pineal GCT in a patient with GIPP and a fluctuating trend of tumor markers, testosterone levels and associated clinical signs, hence prompting the indication for a systematic assessment and a strict monitoring whenever a patient with GnRH-independent precocious puberty shows clinical or radiological markers potentially consistent with a GCT.

## Introduction

GnRH-Independent Precocious Puberty (GIPP) is defined as the development of secondary sexual characteristics before the age of 8 years in girls and 9 years in boys as a consequence of the exposure to either endogenous or exogenous sex steroids, independently of hypothalamic-pituitary-gonadal axis activation.

The etiological *spectrum* of GIPP includes congenital adrenal hyperplasia, familiar male-limited precocious puberty (FMPP), McCune-Albright syndrome, β-human chorionic gonadotrophin (β-hCG)-secreting tumors, sex steroids-secreting tumors, aromatase excess syndrome, hypothyroidism, and exogenous exposure to androgens (1).

Germ cell tumors (GCTs) approximately account for 3% of malignant neoplasms reported in children’s cancer registries (2). GCTs represent a heterogeneous group of tumors, with a high degree of variability in histopathological features, cellular differentiation, tumor markers (β-hCG/alpha-fetoprotein) expression and clinical manifestations. According to the WHO classification system, GCT can be subdivided into germinomas (70% of all GCTs) and non-germinomatous germ cell tumors (NGGCTs, 30%), with the latter including choriocarcinomas, yolk sac tumors, embryonal carcinomas, and mixed tumors. Finally, teratomas represent a separate entity and are classified into “mature” (fully differentiated mesodermic, ectodermic, or endodermic tissue elements) and “immature” (highly undifferentiated tissues) teratomas (3).

The clinical expression of Central Nervous System GCTs depends on the secreting profile, the site and the size of the neoplasm and commonly includes symptoms consistent with raised intracranial pressure, visual disturbances and endocrine disorders. In addition, gender plays a central role, as GCT-induced GIPP does not occur in females.

We hereby report the challenging diagnosis of a pineal GCT in a 6 year-old boy who presented with GIPP and fluctuating levels of β-HCG in serum and cerebrospinal fluid (CSF), demonstrating that also mildly raised tumor markers in the setting of a clinical picture consistent with GIPP should raise the clinical suspicion of a secreting GCT and hence prompt a strict monitoring.

## Case description

A 6.2 year-old boy with an unremarkable previous medical history presented to his local hospital with persistent headaches and a single episode of vomiting. A brain MRI showed a 5 mm bilobed cyst of the pineal gland with a contrast-induced enhancement of the cystic walls and with radiological signs of communicating hydrocephalus.

Four months later the patient was referred to our Pediatric Endocrine and Neuro-Oncology services due to the sudden development of penile enlargement, appearance of pubic hair and growth acceleration in the setting of a pineal lesion. Tanner pubertal staging was genitalia 3, pubic hair 3 and axillary hair 1, with testicular volume of 3 (left) and 4 (right) mL and penile length of 7.5 cm. No skin stigmata suggestive for McCune-Albright syndrome were noticed on examination.

The discrepancy between testicular volume and advanced genitalia staging raised the clinical suspicion of GIPP, which was confirmed by the finding of both baseline and stimulated suppressed gonadotropins with significantly increased testosterone levels (6.85 ng/ml, reference range <0.12 in prepubertal boys). Table 1 summarizes the results of the endocrine tests performed at initial clinical presentation, before any therapeutic intervention was undertaken.

**TABLE 1:** Results of baseline laboratory investigations.

Investigation	Result						Reference range
LH (IU/L)	<0.2						
FSH (IU/L)	0.1						
Testosterone (ng/mL)	6.85						<0.12 (prepubertal)
LHRH stimulation test	*0 min*	*20 min*				*60 min*	
LH (IU/L)	<0.2	0.8				0.8	
FSH (IU/L)	0.6	1.7				2.3	
Urine steroid profile	Qualitatively normal. Modest increase in androgen metabolites						
*LHCGR* gene sequencing	No pathogenic mutation						No pathogenic mutation
Serum alpha-fetoprotein (kU/L)	<1.0						0–5
Serum β–HCG (IU/L)	15.0						<10
CSF alpha-fetoprotein (kU/L)	<1.0						<1.0
CSF β–HCG (IU/L)	4.8						
17-OH-Progesterone (nmol/L)	<1						0–5
Androstenedione (nmol/L)	1.1						0.1–1.1
DHEA-S (μmol/L)	0.7						0.7–5.7
LDH (IU/L)	496						266–500

LH, luteinizing hormone; FSH, follicle stimulating hormone; LHRH, luteinizing hormone releasing hormone; LHCGR, luteinizing hormone/chorionic gonadotropin receptor; β–HCG, β subunit of human chorionic gonadotropin; DHEA-S, Dehydroepiandrosterone Sulfate; LDH, lactate dehydrogenase; CSF, cerebrospinal fluid. In the case presented, total β–HCG levels (defined as HCG *plus* the β–subunit of HCG) were assessed.

A normal genetic sequencing for the LCGHR (luteinizing hormone/choriogonadotropin receptor) gene ruled out FMPP.

**FIGURE 1:**
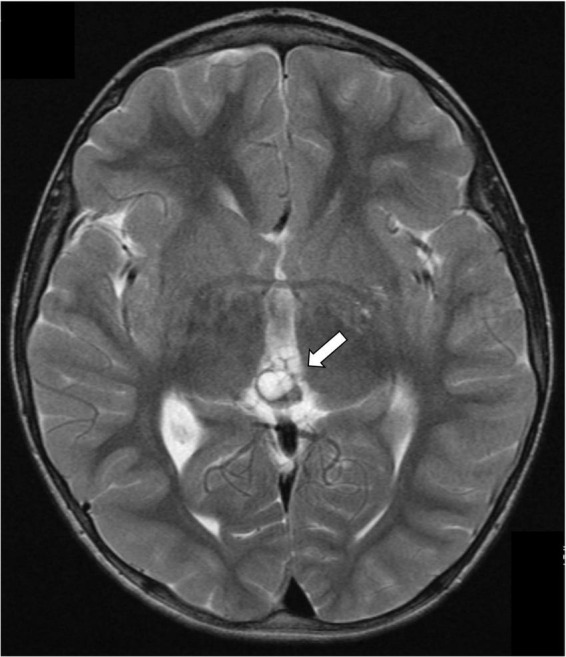
Gadolinium-enhanced brain magnetic resonance highlighting a 5 mm bilobed cyst of the pineal gland (arrow).

Alpha-fetoprotein levels were negative at diagnosis (<1.0 kU/L, normal range: 0–5). However, marginally raised serum β-hCG of 15 IU/L (reference range: 0–10 IU/L, limit of detection of the *assay*: 0.2 IU/L) prompted the indication of a complete oncological work-up, in order to rule out a secreting germ cell tumor: chest CT scan, testicular ultrasound, abdomen and head/spine MRI and ophthalmological evaluation were unremarkable, except for the previously known pineal bilobed cystic lesion (Figure 1). A lumbar puncture showed increased opening pressure (30 cmH_2_O), in keeping with the radiological diagnosis of communicating hydrocephalus. Nevertheless, neither increased AFP or β-hCG concentrations nor tumoral cells were detected in the CSF.

**FIGURE 2:**
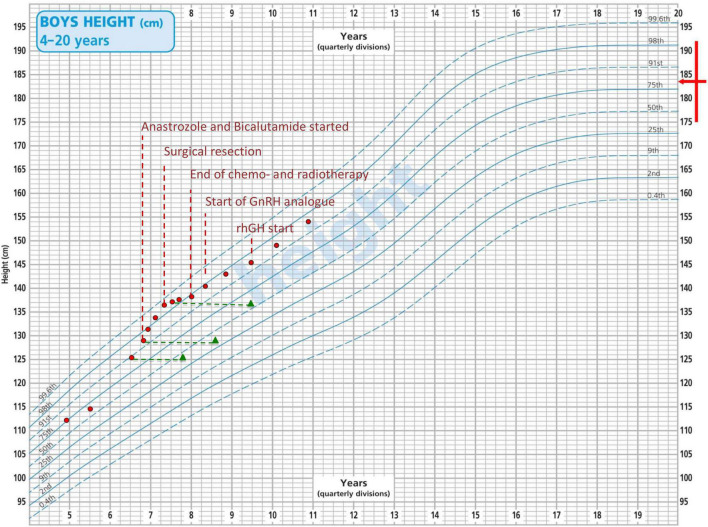
Patient’s growth chart according to the CDC height centiles for reference population. Red dots represent the height recorded at each time point. Green triangles represent patient’s bone age. The red arrow indicates familiar height target.

A close radiological surveillance with 3 monthly MRI scans was undertaken and the pineal cyst remained stable. Monthly blood tests showed a complete and early normalization of both testosterone and serum β-hCG levels until 4 months after initial presentation, when a further progression of pubertal signs, behavioral changes and a remarkable increase in height velocity (Figure 2) corresponded to a significant raise in both testosterone and serum β-hCG levels (15.0 ng/mL and 577 IU/L, respectively) with persistently normal alpha-fetoprotein values and suppressed gonadotropins. A brain MRI was repeated and it showed an increase in the volume of the cyst and radiological signs consistent with an intra-pineal hemorrhage. A second lumbar puncture showed normal AFP (5 kU/L) and raised β-hCG levels (472 IU/L) in CSF. These data supported the diagnostic suspicion of a secreting pineal GCT.

**FIGURE 3:**
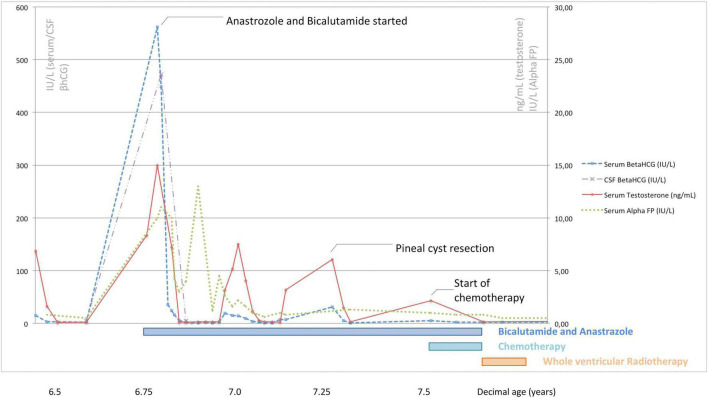
Serum/cerebrospinal β-hCG, serum alpha-fetoprotein, and serum testosterone levels recorded at different time points in relation to the treatments undertaken in the patient described.

Surgery was arranged, but a reassessment of tumor markers immediately before the start of antineoplastic treatment showed an unexpected spontaneous and complete shrinkage of serum and CSF AFP and β-hCG levels and of serum testosterone (Figure 3). The combination of a biologically atypical trend in tumor markers and the finding of CSF-to-serum β-hCG *ratio*<1 (extremely uncommon in patients with intracranial NGGCT) led oncologists to keep surgery on hold and to continue a stringent radiological and biochemical follow-up. Both β-hCG and testosterone levels then remained within the normal ranges for about 2 months.

During this time lag, given the progressive development of pubertal signs and the radiological finding of advanced bone age (Figure 2), the patient was started on a combination treatment with bicalutamide (androgen receptor blocker – starting dose: 50 mg once a day) and anastrozole (aromatase inhibitor – starting dose: 1 mg once a day). From a clinical perspective, pubertal stage did not show any sign of progression and no additional virilization was recorded over a 9-month combined treatment. In addition, parents reported a remarkable improvement of patient’s behavior and a reduction in the frequency of penile erections. However, as showed in Figure 2, height velocity and advanced bone age were not fully reverted despite a progressive increase in the doses administered.

At the age of 7.3 years, a repeat brain MRI showed further increase in the size of the cyst. The patient underwent a surgical excision of the lesion. The histology was consistent with a GCT with a predominant mature teratomatous component (>95% teratoma), as well as a small (<5%) germinoma component, with positive staining for CD117, placental alkaline phosphatase and β-HCG on immunohistochemistry. The tissue was reviewed by three independent pathologists who confirmed the diagnosis.

Following histological confirmation, the patient continued treatment as per the SIOP CNS GCT II trial for localized germinomas with alternating Carboplatin/Etoposide and Ifosfamide/Etoposide. After four cycles of chemotherapy there was no residual disease and the patient received whole ventricular radiotherapy (24 Gy in 15 fractions). Serum levels of testosterone, AFP and β-hCG normalized during treatment (Figure 3), as well as the AFP and β-hCG in CSF, prompting the discontinuation of bicalutamide and anastrozole when the patient was 7.8 years old. At this age, his Tanner stage was genitalia 4, pubic hair 4 and axillary hair 2, with testicular volume of 6 mL bilaterally.

After surgical resection, a remarkable reduction in height velocity was also noted (Figure 2).

Luteinizing hormone and FSH were monitored regularly and remained undetectable until the age of 8.0 years. At the time, the patient presented with a mild increase in testicular volume and a repeat LHRH test confirmed Central Precocious Puberty (CPP; LH peak: 5.0 IU/L, FSH peak 3.8 IU/L, LH/FSH *ratio*>1). Gonadotropin-releasing hormone (GnRH) analog therapy was therefore started.

Upon subsequent auxological follow-up, the finding of a decreased height velocity in a patient who received cranial radiotherapy prompted the prescription of growth hormone (GH) stimulation test, that confirmed a diagnosis of isolated GH deficiency (GH peak after insulin tolerance test: 5.64 ng/mL). The patient was therefore started on recombinant human GH treatment, with a satisfactory catch-up growth.

The patient has been now off-treatment for 3 years and 5 months. He is continuing regular oncological surveillance and shows ongoing complete oncological remission. He is still on GnRH analog and recombinant human GH treatment and he is showing regular height velocity (Figure 2).

**FIGURE 4:**
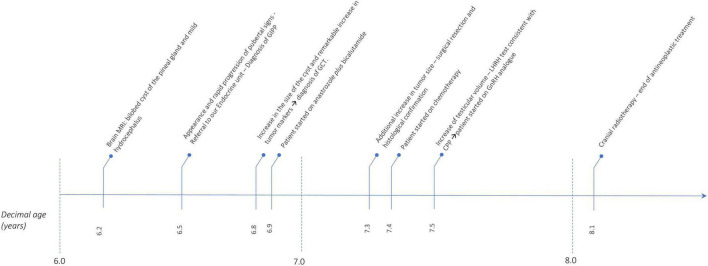
Timeline that summarizes the events reported. MRI, magnetic resonance imaging; GIPP, GnRH independent precocious puberty; GCT, germ cell tumor; CPP, central precocious puberty.

Figure 4 shows the timeline of the events reported.

## Discussion

We reported the challenging process that led to the diagnosis of a pineal mixed GCT in a boy who developed signs of GIPP before the age of 7 years.

Precocious virilization often represents the first sign of a secreting GCT. As hCG and LH share identical α-subunits and similar β-subunits, human chorionic gonadotropin can stimulate testosterone production by Leydig cells as well, causing GIPP.

The therapeutic value of a compound treatment with anastrozole and bicalutamide has been described in patients with familiar male-limited precocious puberty (FMPP) (4, 5). Reiter et al. have demonstrated that this combined therapy arrests the progression of Tanner stage, improves aggressive behavior and remarkably reduces the progression rate of bone age (6). The patient described did not experience any significant adverse events over 9 months of treatment. From a clinical perspective, the combined treatment avoided additional virilization and led to a remarkable improvement in the frequency of erections and episodes of aggressive behavior. Nevertheless, height velocity and advanced bone age were not reverted, hence preventing us from drawing ultimate conclusions about its biological effectiveness. In addition, neither anastrozole nor bicalutamide play a protective role on tumor progression and their function is limited to control peripheral puberty before a resolutive oncological treatment is undertaken.

Pineal cysts frequently represent incidental findings, with reported prevalence ranging from 0.6 to 57% in radiologic and autoptic series and hence requiring neither diagnostic nor therapeutic interventions in most cases (7, 8). It is still debated whether radiological follow-up should be undertaken among asymptomatic patients with cysts sized 10 mm or more (9). In the case reported, despite an overall small size of the cyst (5 mm), a combination of radiological (bilobed appearance, contrast-induced enhancement of the walls, an otherwise unexplained associated hydrocephalus), and clinical findings (GIPP with raised tumor markers, recurrent episodes of vomiting and headaches, raised intracranial pressure) prompted a strict radiological and biochemical follow-up that resulted in the histological diagnosis of secreting pineal GCT.

It is not uncommon that patients with CNS GCT experience a time lag before the tumoral source can be identified. For instance, Crawford et al. reported delays in diagnosis ranging between 7 months and 3 years in a series of 30 patients with GCT (10). Similarly, Sethi et al. reported delays of at least 6 months to diagnose 38 out of 70 patients (54%) with GCT (11). In our patient, 7 months elapsed between the initial presentation with GIPP and the diagnosis of a secreting GCT.

The formal diagnosis of germ cell tumor is based whenever possible on histology. However, when the radiological appearances are highly suggestive of a GCT (e.g., pineal or suprasellar location) and the tumor markers are significantly elevated in blood and/or CSF, it is also possible to make a clinical-radiological diagnosis of a secreting GCT and proceed with anticancer treatment.

Secreting GCT are generally accepted to be malignant NG-GCT (12). However, germinomas can also be associated with elevation of total β-hCG due to the presence of syncytiotrophoblastic cells (13, 14). The optimal cut-off of β-hCG levels to discriminate germinomas from NG-GCT remains controversial and no agreement can be found among different consortia. For instance, the SIOP CNS GCT II trial in Europe establishes as diagnostic tumor markers in serum and/or CSF for NG-GCT: AFP > 25 ng/ml and/or β-HCG > 50 IU/L (NCT01424839); whereas the ACNS1123 trial in the United States suggests AFP > 10 ng/ml and/or β-HCG > 100 IU/L (NCT01602666).

The prognostic value of β-HCG levels in germinomas is also unclear. Some studies suggest that germinoma patients with elevated β-HCG levels are at increased risk of relapse and show worse survival rates than those with normal β-hCG levels, whereas, other Authors challenge this hypothesis (13, 15).

Noteworthy, our case highlights the potential highly fluctuating trend of β-hCG levels in the setting of a GCT-induced GIPP. The diagnostic contribution of β-hCG levels at first biochemical evaluation, just slightly above the reference range, may have been underestimated. Superimposable conclusions have been drawn by Arya and Davies, who reported a similar fluctuating trend in tumor markers in a 5-year child with GCT-induced GIPP (16).

In our case, the combination of clinical and biochemical findings consistent with GIPP and the radiological appearance of the pineal gland prompted a systematical monitoring program that allowed us to detect several phases of remarkable contemporaneous rise in testosterone and serum/CSF β-HCG levels, followed by an unexpected spontaneous decrease. We hypothesized that recurrent hemorrhages occurring within the tumor may have caused the sudden and unpredictable release of tumor markers in the bloodstream. In addition, as the CNS is an immunological sanctuary, hemorrhages may have disrupted the integrity of the brain-blood-barrier, resulting in the exposure of otherwise undetected tumor antigens. It is likely that the immune system may have played a contributory role in modulating tumor growth and, accordingly, the release of β-HCG in the CSF and blood stream, resulting in fluctuating circulating levels. This hypothesis is drawn from the experience gathered in patients with testicular germinomas. Testes are immunologically privileged sites, but the exposure of neoplastic antigens following orchiectomy or diagnostic surgery often results in an immunologic over-reaction promoted by CD8+ and CD4+ lymphocytes (17).

Only repeated withdrawals allowed the detection of β-hCG peaks that finally supported the diagnosis of a secreting GCT. As the fluctuations of tumor markers in the case presented were completely unpredictable, no clear recommendations about the timing of biochemical monitoring can be provided. Nevertheless, β-HCG and testosterone flares were always followed by overt worsening of penile erections and aggressive behavior, hence supporting a clinically guided biochemical assessment.

Finally, the onset of CPP following GIPP may have different explanations. Firstly, intermittently raised testosterone levels experienced during GIPP can trigger CPP, irrespectively of the endogenous or exogenous source of androgens. Secondly, the patient underwent cranial radiation, that has been demonstrated as the most effective therapy in patients with malignant germ cell tumors (18). As a result of the widely described detrimental role of radiotherapy on the GABAergic hypothalamic neurons inhibiting gonadotropin precocious activation, this may result in CPP. Thirdly, a possible association between pineal cysts and central precocious puberty has been described: the precise mechanism still needs to be clarified, but is has been suggested that the pineal gland plays a direct inhibitory role on gonadotropin release while melatonin secretion may prevent a precocious activation of hypothalamic-pituitary-gonadal axis (19, 20). A pineal cyst may disrupt both these mechanisms and involve CPP.

In conclusion, we hereby documented the case of a child with histologically confirmed pineal teratoma/germinoma with markedly raised β-hCG levels which normalized spontaneously. Hence providing evidence to challenge the concept that β-hCG levels can effectively discriminate germinomatous and non-germinomatous GCT and prompting the indication for a strict monitoring of biochemical tumor markers whenever a patient with GIPP shows a clinical or radiological marker potentially consistent with a GCT.

Accordingly, it is not uncommon that patients with CNS GCT experience a remarkable time lag before the tumoral source is identified and the diagnostic suspicion is verified. In the case presented, a combined treatment with anastrozole and bicalutamide was administered as a bridge therapy in the attempt to control pubertal progression before histological confirmation and thus before antineoplastic treatment was undertaken.

## Data availability statement

The raw data supporting the conclusions of this article will be made available by the authors, without undue reservation.

## Ethics statement

Ethical review and approval was not required for the study on human participants in accordance with the local legislation and institutional requirements. Written informed consent to participate in this study was provided by the participants’ legal guardian/next of kin. Written informed consent was obtained from the minor(s)’ legal guardian/next of kin for the publication of any potentially identifiable images or data included in this article.

## Author contributions

AC and AA conceived the idea of describing the present case report and critically revised its contents. AC drafted the manuscript and produced the pictures. AA critically revised the contents of the manuscript and approved the final outcome. Both authors contributed to the article and approved the submitted version.
